# Biomechanical Stimulation of Muscle Constructs Influences Phenotype of Bone Constructs by Modulating Myokine Secretion

**DOI:** 10.1002/jbm4.10804

**Published:** 2023-08-15

**Authors:** Harshini Suresh Kumar, Edwina N. Barnett, John L. Fowlkes, Evangelia Kalaitzoglou, Ramkumar T. Annamalai

**Affiliations:** ^1^ Department of Biomedical Engineering University of Kentucky Lexington KY USA; ^2^ Barnstable Brown Diabetes Center Lexington KY USA; ^3^ Department of Pediatrics University of Kentucky Lexington KY USA

**Keywords:** BONE, DIABETES, EXERCISE, GELATIN, MICROGELS, MUSCLE, MYOSTATIN

## Abstract

Diabetes is a chronic metabolic disorder that can lead to diabetic myopathy and bone diseases. The etiology of musculoskeletal complications in such metabolic disorders and the interplay between the muscular and osseous systems are not well understood. Exercise training promises to prevent diabetic myopathy and bone disease and offer protection. Although the muscle‐bone interaction is largely biomechanical, the muscle secretome has significant implications for bone biology. Uncoupling effects of biophysical and biochemical stimuli on the adaptive response of bone during exercise training may offer therapeutic targets for diabetic bone disease. Here, we have developed an in vitro model to elucidate the effects of mechanical strain on myokine secretion and its impact on bone metabolism decoupled from physical stimuli. We developed bone constructs using cross‐linked gelatin, which facilitated osteogenic differentiation of osteoprogenitor cells. Then muscle constructs were made from fibrin, which enabled myoblast differentiation and myotube formation. We investigated the myokine expression by muscle constructs under strain regimens replicating endurance (END) and high‐intensity interval training (HIIT) in hyperglycemic conditions. In monocultures, both regimens induced higher expression of *Il15* and *Igf1*, whereas END supported more myoblast differentiation and myotube maturation than HIIT. When co‐cultured with bone constructs, HIIT regimen increased *Glut4* expression in muscle constructs more than END, supporting higher glucose uptake. Likewise, the muscle constructs under the HIIT regimen promoted a healthier and more matured bone phenotype than END. Under static conditions, myostatin (*Mstn*) expression was significantly downregulated in muscle constructs co‐cultured with bone constructs compared with monocultures. Together, our in vitro co‐culture system allowed orthogonal manipulation of mechanical strain on muscle constructs while facilitating bone‐muscle biochemical cross‐talk. Such systems can provide an individualized microenvironment that allows decoupled biomechanical manipulation, help identify molecular targets, and develop engineered therapies for metabolic bone disease. © 2023 The Authors. *JBMR Plus* published by Wiley Periodicals LLC. on behalf of American Society for Bone and Mineral Research.

## Graphical abstract

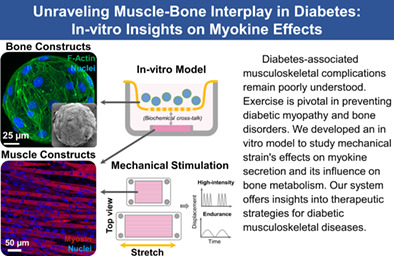

## Introduction

Diabetes mellitus is a collection of metabolic illnesses defined by long‐term hyperglycemia caused by defects in insulin secretion and action.^(^ ^1^, ^2^, ^3^ ^)^ Diabetes can affect every organ system in the human body, and the extent of pathophysiology depends on the severity and duration of the disease. Diabetes also leads to major musculoskeletal complications, including diabetic myopathy and diabetic bone disease. Diabetic myopathy is characterized by skeletal muscle loss and impaired function secondary to muscular inflammation, ischemia, hemorrhage, infarction, necrosis, fibrosis, and fatty atrophy.^(^ ^4^ ^)^ Diabetic bone disease is characterized by poor bone quality, leading to increased fracture risk, osteoporosis, and delayed bone healing.^(^ ^5^ ^)^ Several factors such as severity and duration of diabetes, age at diagnosis, lack of resistance to endogenous insulin, and micro‐ and macrovascular diabetes complications have been implicated in the development of diabetic myopathy^(^ ^6^, ^7^ ^)^ and diabetic bone disease.^(^ ^8^, ^9^ ^)^ Additionally, treatment of diabetes with oral or injectable hypoglycemic agents can have negative effects on the musculoskeletal system.^(^ ^9^, ^10^ ^)^ For diabetes‐mediated musculoskeletal pathologies such as myopathy, symptomatic alleviation is the primary treatment that involves pain management, rest, and anti‐inflammatory medicines.^(^ ^11^ ^)^ Pharmacotherapies using disease‐modifying agents such as anti‐myostatin peptides, *STAT3* inhibitors, anabolic androgenic steroids, *IGF‐1*, *PPAR* agonists, and antisense oligonucleotides have been studied to treat diabetic myopathy.^(^ ^10^, ^12^, ^13^, ^14^ ^)^ Likewise, antiresorptive or anabolic medications containing bisphosphonates could be useful in preventing diabetes‐related fractures.^(^ ^15^ ^)^ Although these pharmaceutical agents effectively ameliorate specific symptoms, they often cause off‐target effects.

Physical exercise has shown beneficial effects, including improved glucose metabolism and insulin sensitivity and is often recommended along with pharmacotherapies for the clinical management of diabetes.^(^ ^16^ ^)^ An acute bout of exercise increases muscle glucose uptake efficiency, and chronic exercise training enhances mitochondrial biogenesis, increasing the expression levels of glucose transporter proteins and various metabolic genes.^(^ ^17^ ^)^ Furthermore, the contractions of muscles can have a positive impact on the metabolism of other tissues and organs, including bone. For many years, scientists have explored pathways induced by muscle contractions and how they can influence the bone phenotype in both normal and pathological conditions. Thus, investigating the interplay between different tissues and their physiological responses to muscle contractions may provide novel insights into the pathogenesis of musculoskeletal diseases and lead to the development of more effective treatments.

The muscle‐bone interaction is primarily mechanical, with muscle imposing contractile forces on bone tissue.^(^ ^18^ ^)^ But skeletal muscle also acts as an endocrine organ by secreting factors called myokines that are implicated in various physiological and pathological mechanisms in other organs, including bone.^(^ ^19^, ^20^, ^21^ ^)^ Factors related to exercise training such as duration, intensity, muscle mass, and endurance capacity determine the type and magnitude of myokine secretion from skeletal muscle.^(^ ^22^, ^23^ ^)^ A subset of myokines produced by skeletal muscle contraction can positively influence bone formation and regulate glucose uptake rate. Under hyperglycemic conditions, the synthesis and secretion of several myokines are altered.^(^ ^24^, ^25^, ^26^ ^)^ It has been hypothesized that this diabetic‐mediated imbalance in myokine secretion plays a crucial role in developing diabetic myopathy and bone diseases.^(^ ^27^, ^28^ ^)^ Myostatin and irisin are key myokines known to promote osteoclastogenesis and osteoblast differentiation, respectively.^(^ ^29^, ^30^ ^)^ Although myostatin is critical for osteoclast development, it can have detrimental effects on bone mass by reducing bone formation and increasing bone resorption.^(^ ^21^ ^)^ Remarkably, weight‐bearing exercises are shown to inhibit myostatin expression and reduce the blood levels of alkaline phosphatase (ALP) and tartrate‐resistant acid phosphatase (TRAP) in a rat T1D model.^(^ ^31^ ^)^ On the other hand, irisin can upregulate osteogenic genes such as *RUNX2*, Osterix (*OSX*), *ATF4*, β‐catenin (*CTNNB1*), alkaline phosphatase (*ALP*), and type I collagen (*COL1A1*) in osteopotent cells. Follistatin, an endogenous inhibitor of myostatin, is shown to increase after resistance exercise. Resistance exercise activates mTORC1‐mediated increase in miR‐1, which decreases myostatin activity by secreting follistatin.^(^ ^32^ ^)^ Some anti‐inflammatory cytokines, including IL‐10, IL‐1Ra, and soluble receptors of the tumor necrosis factor (TNF) I and II, were also shown to increase after acute aerobic exercise.^(^ ^33^ ^)^ However, the underlying cellular pathways regulating the muscle secretome during exercise training and its protective effects on bone physiology and function are poorly understood. Hence, it is crucial to understand the effect of exercise on myokine secretion pattern and its effect on the bone to determine molecular targets for developing therapies for diabetes‐mediated myopathy and bone disease.

**Fig. 1:**
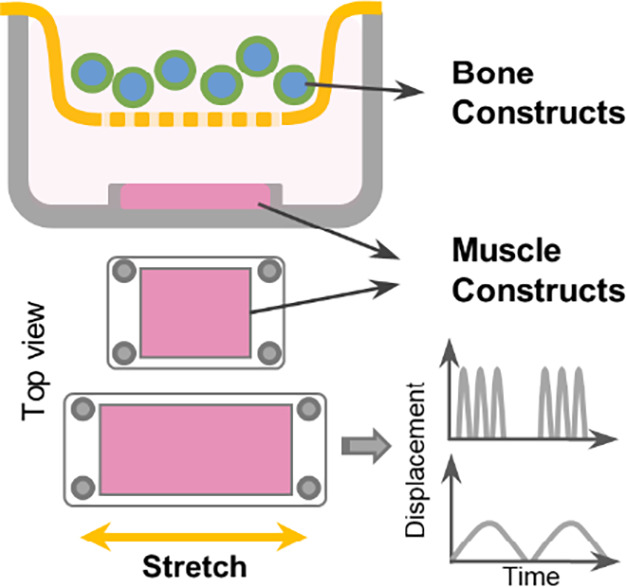
Muscle‐bone cross‐talk in diabetes. The schematic shows the co‐culture system that was set up to elucidate the impact of muscle exercise on bone metabolism. We fabricated bone and muscle constructs using natural polymers and configurations, allowing cells to exhibit their innate tissue‐specific phenotype.

Uncoupling the effects of biophysical and biochemical stimuli on the adaptive response of bone during exercise training is challenging using animal models. Here, we have developed an in vitro culture system to elucidate the effects of mechanical strain on myokine secretion and its impact on bone metabolism decoupled from physical stimuli. We fabricated bone and muscle constructs using natural polymers and configurations that allow the cells to exhibit their innate tissue‐specific phenotype. Our system provides an individualized microenvironment and allows decoupled biomechanical manipulation, which is not achievable in animal models. Then we developed an in vitro culture apparatus using these engineered constructs that allowed orthogonal manipulation of mechanical strain on muscle constructs while facilitating biochemical cross‐talk between bone and muscle constructs, as shown in Figure 1. We hypothesize that the type and intensity of mechanical strain regulate the myokine secretion, which influences myotube and osteoblast phenotype and function in a hyperglycemic microenvironment. We investigated the secretion of myotubes under endurance (END) and high‐intensity interval (HIIT) strain regimens under hyperglycemic conditions and their effects on bone metabolism and vice versa. The effect of myostatin, a key myokine implicated in several diabetes‐driven pathologies, on bone constructs was investigated. The function of follistatin to revert the adverse effect of myostatin on bone cells was also explored. Overall, our studies elucidate the effects of biophysical and biochemical stimuli on muscle secretome and its corresponding impact on bone phenotype and vice versa in a hyperglycemic environment. Our work is crucial for identifying novel therapeutic targets and help develop engineered therapies for various diabetes‐related complications.

## Materials and Methods

### Fabrication of bone constructs

We used cross‐linked gelatin microspheres and seeded them with preosteoblasts to fabricate bone constructs, as shown previously.^(^ ^34^, ^35^ ^)^ Gelatin is obtained from partially hydrolyzed collagen retaining RGD motifs for cell attachment, biocompatibility, and osteoconductivity.^(^ ^36^ ^)^ In addition, the microsphere format provides a 3D microenvironment with physical cues that can promote osteogenic differentiation of progenitor cells.^(^ ^37^ ^)^ To fabricate bone constructs, we made 3D microgels (~150 μm dia.) from solubilized gelatin through a simple water‐in‐oil emulsification process and cross‐linked them using genipin (Wako, Richmond, VA, USA), as described.^(^ ^34^, ^35^ ^)^ Briefly, the 6 wt.% gelatin solution (Porcine skin, Type A, Sigma, St. Louis, MO, USA) in deionized (DI) water was added to a beaker containing stirred polydimethylsiloxane (PDMS) at 37°C and emulsified for 5 minutes. The mixture was stirred at 450 rpm using a spiral propeller blade, and the emulsion was then cooled to 4°C for 30 minutes using an ice bath. The emulsion was then centrifuged at 175*g* for 5 minutes to separate the gelatin microgels from PDMS. The pellet was washed thrice with 10 mM PBS (Gibco, Thermo Fisher Scientific, Waltham, MA, USA) containing 1% TWEEN 20 (Sigma, PBS‐T20) and suspended in genipin (1 wt.% in 1X PBS) for 48 hours. The cross‐linked microgels were then washed in 100% ethanol to remove excess genipin. The microgels were filtered using nylon sieves to obtain the desired size range of 100 to 150 μm in diameter. To fabricate bone constructs, the microgels were sterilized with ethanol overnight and seeded with murine preosteoblast cell line (MC3T3, Riken BRC, Tsukuba, Japan) at a density of 4 × 10^6^ cells/mg dry mass of microgels. The cells were cultured in α‐modified Eagle's medium (α‐MEM, Gibco, 1 g/L or 5.5 mM of glucose) supplemented with 10% v/v FBS, 1X antibiotic‐antimycotic solution (AA, Gibco), and 0.2% normocin (InvivoGen, San Diego, CA, USA) and maintained in a humidified CO_2_ incubator. To induce osteogenic differentiation, α‐MEM supplemented with 10% FBS, 1% 20 mM ascorbic acid 2 phosphate, 1% 1 M β glycerol phosphate was added to the microgel suspension and cultured for 5 days.

### Fabrication of muscle constructs

The muscle constructs were fabricated by seeding myoblasts in 3D fibrin hydrogels. Fibrin facilitates muscle cell migration and proliferation without spatial inhibition, enabling them to self‐organize into myotubes,^(^ ^38^ ^)^ and has been used to regenerate skeletal muscles and treat muscular dystrophies. Further, fibrin is known to bind to angiogenic factors, including vascular endothelial growth factor (VEGF), basic fibroblast growth factor‐2 (bFGF‐2), and insulin‐like growth factor‐1 (IGF) that participate in myogenesis.^(^ ^39^ ^)^ To fabricate muscle constructs, we used murine myoblasts (C2C12, ATCC) encapsulated in fibrin hydrogel. To make the fibrin hydrogel, fibrinogen stock (4 mg of clottable protein/mL of DMEM, bovine plasma, type I‐S, Sigma) was filter‐sterilized and mixed with myoblasts suspension. Thrombin (Sigma) was added to cleave the fibrinogen molecule and form 3D fibrin gel. The final concentration of the mixture was 2.5 mg/mL of fibrinogen/fibrin, 1 U/mL of thrombin, and a cell density of 2 × 10^6^ cells/mL. The mixture was then quickly cast into specially molded 12 × 12 mm PDMS wells and placed in a humidified incubator for 45 minutes to allow complete gelation. The cells were supplied with growth media DMEM‐GlutaMax (4.5 g/L or 25 mM of glucose, 10% FBS, 1X AA, and 0.2% normocin) for 48 hours and switched to differentiation media (DMEM‐GlutaMax) supplemented with 2% horse serum (Sigma), 1% AA, and 0.2% normocin) and differentiated for 5 days. Media was replenished daily until the mature myotubes formed were verified. Fully matured muscle constructs were used for characterization and co‐culture experiments.

### Strain regimens of muscle constructs

The muscle constructs were subjected to linear strain using Cytostretcher (CuriBio, Seattle, WA, USA). Muscle constructs with mature myotubes were subjected to acute high‐intensity strain (10% strain at 1/30 Hz for 1‐hour bouts, repeated over 8 hours with 5‐hour intervals) or chronic moderate strain (1%–2% strain at 1/30 Hz in bouts of 6 hours for 24 hours with 6‐hour intervals) mimicking high‐intensity interval training (HIIT) and endurance (END) exercise, respectively. Static cultures served as a control. For the co‐culture experiments, the bone constructs were kept above the muscle constructs either using membrane‐bottom rectangular trans wells (5 × 5 mm) or directly on top of the muscle constructs to allow biochemical communications between the constructs without any strain transfer. We used DMEM with high glucose (4.5 g/L or 25 mM of glucose) for all co‐culture studies to emulate diabetic conditions.

### Immunofluorescence and confocal microscopy

Bone and muscle samples collected at different time points were fixed in 10% buffered formalin (Thermo Fisher Scientific), permeabilized with 0.1% Triton X‐100 (Sigma) in 1X PBS, and blocked with 1% bovine serum albumin (BSA) in 10 mM PBS. The cells were then stained for cytoskeletal F‐actin using phalloidin conjugated with Alexa fluor 488 or Alexa fluor 594 (Invitrogen, Carlsbad, CA, USA) and DAPI (Life Technologies, Carlsbad, CA, USA) as nuclear counterstaining. Additionally, the muscle construct was stained for myosin heavy chain to characterize myotube formation. The muscle construct was incubated with the primary antibody for myosin heavy chain (Sigma M4276, 1:400 dilution) for 2 hours at room temperature. Then the samples were washed thrice in PBS‐T20, and a goat anti‐mouse IgG1 secondary antibody conjugated with Alexa fluor 488 (Invitrogen A21121, 1:500) was added and incubated for 1 hour at room temperature. Then the samples were mounted in ProLong Gold anti‐fade media (for 2D cultures only), and confocal stacks were acquired using Nikon (Tokyo, Japan) A1R inverted confocal microscope.

### Electron microscopy

Bone and muscle samples collected at different time points were fixed with 3% glutaraldehyde for a minimum of 1 hour. Constructs were then washed in PBS and DI water and suspended in 1% osmium tetroxide for 16 hours at 4°C. Then the samples were washed in DI water, dehydrated using an ethanol series (30%, 50%, 70%, 80%, 90%, 96%, 100%), and freeze‐dried overnight. The dried samples were then mounted to stubs containing carbon adhesive, coated with 5 nm layer of platinum using a sputter coater (Leica [Buffalo Grove, IL, USA] ACE600). Then the coated samples were imaged under a scanning electron microscope (FEI Quanta 250, Thermo Fisher Scientific).

### Compression testing

The stiffness of the genipin cross‐linked gelatin hydrogel was determined using the compression testing of cylindrical blocks of the samples using a universal mechanical testing machine (Instron [Norwood, MA, USA] 6800). Briefly, the gelatin‐genipin hydrogel was cast between two parallel glass plates separated by a 5 mm spacer. Using an 8 mm biopsy punch, samples were punched out and compressed at a rate of 2 mm/min to obtain the stress–strain curve. Young's modulus was then calculated from the slope of the linear portion of the curve between 5% and 15% strain.

### Quantification of secreted myokines

Secreted myostatin (MSTN) and insulin‐like growth factor (IGF‐1) were measured in conditioned media from muscle constructs that were subjected to static or strain regimens by ELISA as per the manufacturer's recommendations (GDF‐8/Myostatin Quantikine ELISA Kit, R&D Systems/Bio‐Techne [Minneapolis, MN, USA], catalog #DGDF80, and Mouse IGF‐I ELISA Kit, Millipore/Sigma [Burlington, MA, USA], catalog # RAB0229). The conditioned media from the static control and the strain groups were collected immediately after the last bout of the mechanical strain regimen and kept in −20°C until further analysis. Follistatin was measured using a MILLIPLEX MAP Mouse Angiogenesis/Growth Factor Magnetic Bead Panel (Millipore, catalog #MAGPMAG‐24 K‐1) and follistatin‐like 1 (FSTL‐1), osteonectin (SPARC), irisin (FNDC5), oncostatin M (OSM), interleukin 15 (IL‐15), and interleukin‐6 (IL‐6) were measured using a MILLIPLEX Mouse Myokine Magnetic Bead Panel (Millipore, catalog #MMYOMAG‐74K‐10) following manufacturer's recommendations.

### Myostatin and follistatin treatment

Preosteoblast cells (MC3T3) cultured in tissue culture plates were differentiated for 5 days and treated with myostatin at 100 ng/mL of media (788‐G8‐010/CF R&D Systems), follistatin at 100 ng/mL of media (769‐FS‐025/CF, R&D Systems), and a cocktail of myostatin and follistatin (50 ng/mL each) for 2 or 7 days. The cells were detached and analyzed for the expression of prominent osteogenic genes.

### Quantitative gene expression

The samples were collected in TRIzol reagent (Invitrogen), and the total RNA was isolated following the manufacturer's protocol. An amount of 100 ng of RNA was used for gene expression assay. Real‐time quantitative polymerase chain reaction was carried out in duplicates with reaction volumes of 10 μL using TaqMan probes (Advanced Bioscience, Rockville, MD, USA) for myogenic genes *Igf‐1* (insulin growth factor 1 Mm00439560_m1), *Fgf‐2* (fibroblast growth factor Mm00433287_m1), *Mstn* (myostatin, Mm01254559_m1), *Fndc‐5* (irisin, Mm01181543_m1), *Il‐15* (interleukin‐15, Mm00434210_m1), *Myog* (myogenin, Mm00446194_m1), *Myod1* (myoblast determination protein‐1 Mm00440387_m1), *Fst* (follistatin, Mm00514982_m1), *Glut4* (glucose transporter type 4, Mm00436615_m1), *Ppia* (peptidylprolyl isomerase, Mm02342430_g1) and osteogenic—*Alp1* (alkaline phosphatase 1, Mm00475834_m1), *Col1a1* (collagen type I alpha 1 Mm00801666_g1), *Runx‐2* (Runt‐related transcription factor 2, Mm00501584_m1), *Bglap* (osteoclacin, Mm03413826_mH), *Spp1* (osteopontin, Mm00436767_m1), *Ibsp* (integrin binding sialoprotein, Mm00492555_m1), and *Sp‐7* (osterix, Mm04209856_m1). *Gapdh* (glyceraldehyde‐3‐phosphate dehydrogenase, Mm99999915_g1) and *Actb* (actin beta, Mm02619580_g1) were used as internal controls. A SuperScript III Platinum One‐Step qRT‐PCR Kit (Thermo Fisher Scientific) was used for reverse transcription, and the gene expression study was performed using QuantStudio 3 real‐time PCR system (Thermo Fisher Scientific).

### Statistics

All experiments were performed in at least triplicates. Statistical comparisons were made using Student's *t* test and one‐way ANOVA with a 95% confidence limit. Dunnett test was performed to compare the treated groups with a single control group and the Tukey test to compare independent groups. Differences with *p* < 0.05 were considered statistically significant.

## Results

### Bone constructs support osteogenic differentiation of progenitor cells

**Fig. 2:**
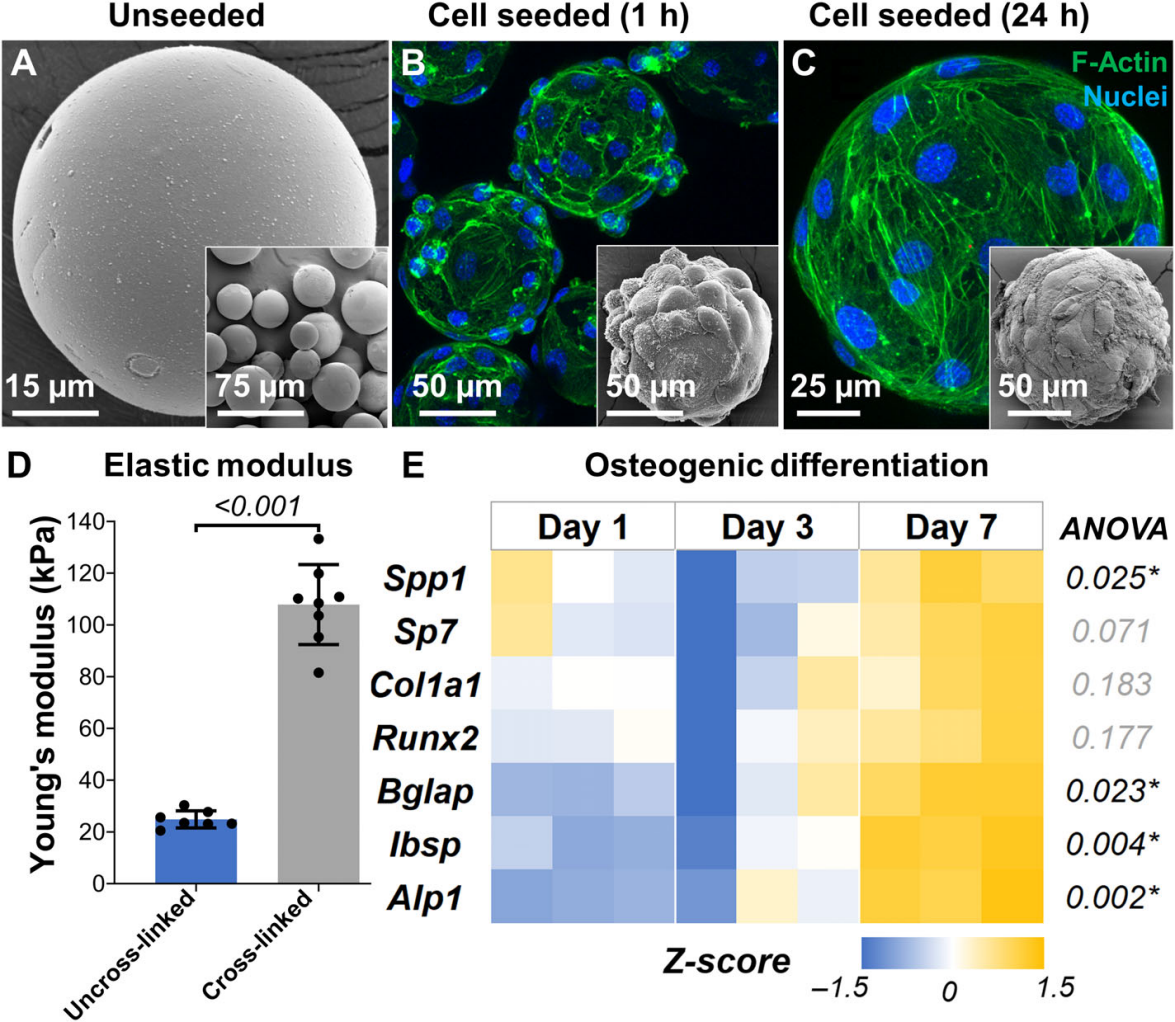
Fabrication and characterization of bone constructs. (*A*) Scanning electron micrograph of lyophilized microgels showing smooth surface morphology. Confocal stacks of preosteoblasts on microgel surface (*B* **)** 1 hour after seeding and (*C*) 24 hours after seeding. The insets show electron micrographs of the corresponding samples. (*D*) Elastic modulus of cross‐linked gelatin microgels compared with uncross‐linked gelatin. *n* = 7 for all groups. (*E*) Heat map showing the relative expression of osteogenic genes over time by osteoprogenitor cells seeded on microgels. All gene expressions were normalized to a no‐treatment control at day 1 and housekeeping gene Gapdh expression. *n* = 3 for all groups.

We fabricated cross‐linked gelatin microgels with a size range of 150 to 250 μm (Fig. 2*A*), sterilized them, and seeded them with preosteoblasts (MC3T3 cells). The cells readily attached to the microgel surface within an hour of seeding (Fig. 2*B*) and exhibited an osteoblast phenotype with rich actin cytoskeleton by 24 hours (Fig. 2*C*). The progenitor cells proliferated on the surface of the microgel, and when maintained for an extended period, they tended to form aggregates in suspension cultures. Using compression testing, we found that cross‐linked gelatin has an elastic modulus of 111.61 ± 12.1 kPa, whereas the uncross‐linked hydrogels had only 24.8 ± 3.3 kPa (Fig. 2*D*). Then, the osteogenic potential of the microgels was assessed using long‐term cultures. The osteoblast progenitors were seeded on microgels, maintained in growth and osteogenic conditions, collected at various time points, and analyzed for prominent osteogenic marker expression. In osteogenic culture conditions, the progenitor cells differentiated and upregulated the expression of prominent osteogenic genes within a week (Fig. 2*E*). There was a significant increase in the expression of *Alp1* (alkaline phosphatase), *Bglap* (osteocalcin), *Ibsp* (bone sialoprotein), and *Spp1* (osteopontin) by day 7 (see Table 1 for groupwise comparison). There was no significant change in osteogenic gene expression under growth conditions (Supplemental Fig. S1). These results suggest that the cross‐linked gelatin constructs were conducive to osteogenesis and appropriate for fabricating bone constructs.

**Table 1:** Osteogenic Differentiation of Progenitor Cells Seeded on Cross‐Linked Gelatin Microgels

Genes	ANOVA	Day 1 vs. day 3	Day 1 vs. day 7	Day 3 vs. day 7
*Alp1*	0.002*	0.329	0.002*	0.012*
*Col1a1*	0.183	0.622	0.512	0.163
*Runx2*	0.177	0.734	0.414	0.162
*Bglap*	0.023*	0.832	0.026*	0.054
*Spp1*	0.025*	0.133	0.375	0.022*
*Ibsp*	0.004*	0.741	0.004*	0.010*
*Sp7*	0.071	0.345	0.403	0.061

*Note*: Tukey multiple comparisons test (*p* value).

* Indicates *p* < 0.05.

### Muscle constructs supported myogenesis and myotube formation

**Fig. 3:**
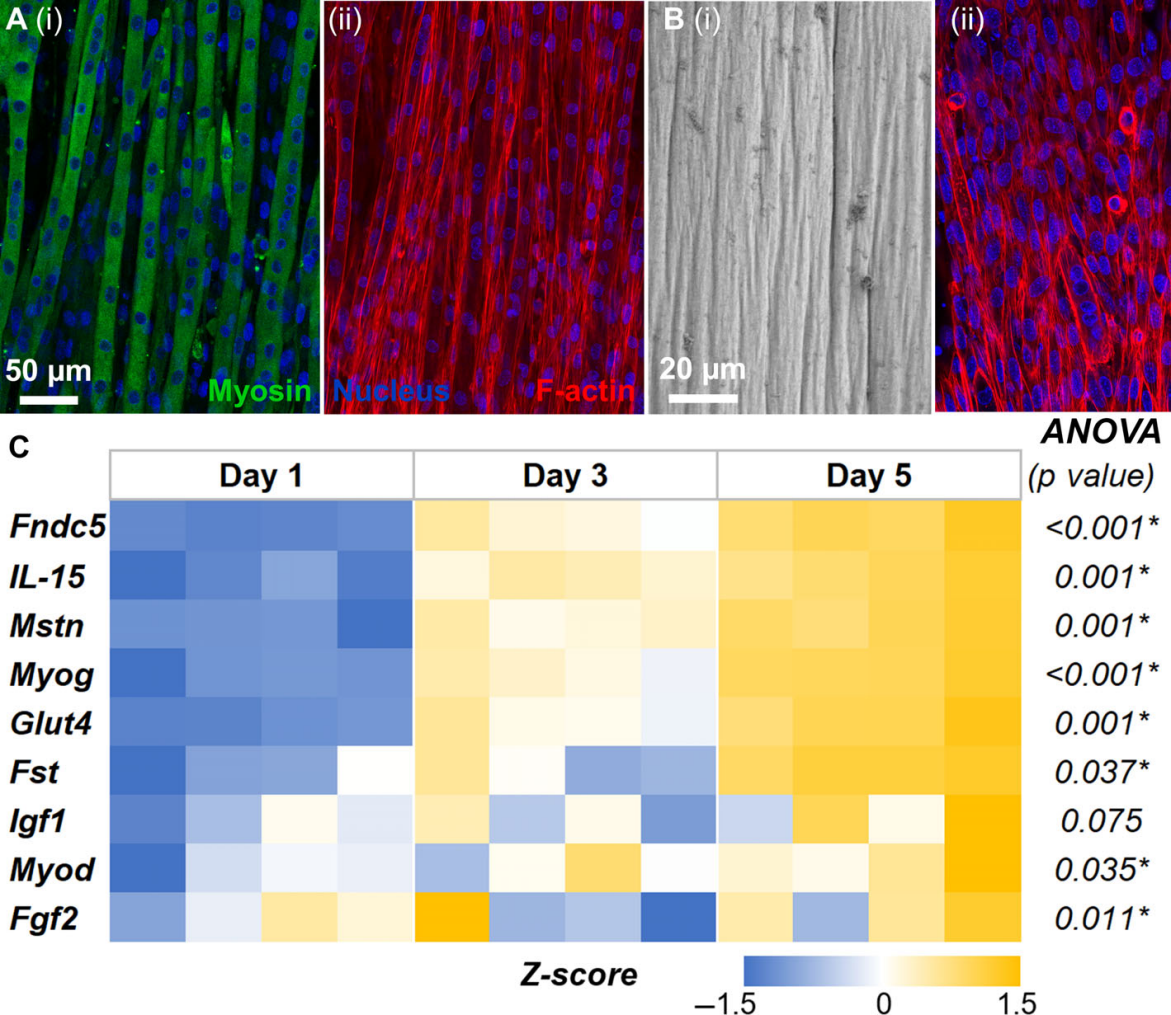
Fabrication and characterization of skeletal muscle constructs. (*A*) Confocal image of myoblasts differentiated into multinucleated myotubes expressing myosin in tissue culture plastic. (*B*) Scanning electron micrograph of myobundles in 3D fibrin hydrogels. (*C*) Temporal gene expression profiles of the myoblasts undergoing differentiation in fibrin hydrogel showed significant changes in the expression levels of prominent skeletal muscle genes. All gene expressions were normalized to a no‐treatment control at day 1 and housekeeping gene Ppia expression. *n* = 4 for each group.

We created muscle constructs by seeding murine myoblasts (C2C12 cells) in fibrin hydrogels and differentiating them to form myotubes through serum starvation. The differentiation protocol was optimized by culturing myoblasts in tissue culture plastics, which showed that 5‐day serum starvation resulted in well‐defined multinucleated myotubes (Fig. 3*A*). The differentiation of myoblasts yielded myobundles when seeded in 3D fibrin hydrogels (Fig. 3*B*). Further, the temporal gene expression profiles of the myoblasts undergoing differentiation in fibrin hydrogel showed significant changes in the expression levels of prominent skeletal muscle genes (Fig. 3*C*). Specifically, myoblast determination protein 1 (*Myod1*) and myogenin (*Myog*) expression was upregulated on day 3. The expression of myogenin (*Myog*) continued to be significantly upregulated until day 5 (fourfold increase, *p* < 0.001), indicating myoblast differentiation to multinucleated myotubes. Likewise, there was a significant upregulation of glucose transporter 4 (*Glut4*), *Mstn*, *Fndc5*, and interleukin‐15 (*Il‐15*) over the 5 days, indicating myoblast differentiation in the fibrin constructs. No significant changes in myoblast determination protein 1 (*Myod1*), insulin‐like growth factor 1 (*Igf1*), and fibroblast growth factor 2 (*Fgf2*) were noticed from day 3 to day 5 (see Table 2 for groupwise comparison).

**Table 2:** Myoblast Differentiation

Genes	ANOVA	Day 1 vs. day 3	Day 1 vs. day 5	Day 3 vs. day 5
*Fndc5*	<0.001*	0.006*	<0.001*	0.012*
*IL‐15*	0.001*	0.056	0.001*	0.048*
*Mstn*	0.001*	0.038*	0.001*	0.064
*Myog*	<0.001*	0.005*	<0.001*	0.002*
*Glut4*	0.001*	0.162	0.001*	0.017*
*Fst*	0.037*	0.155	0.601	0.033*
*Igf1*	0.075	0.071	0.766	0.206
*Myod*	0.035*	0.033*	0.695	0.121
*Fgf2*	0.011*	0.011*	0.048*	0.636

*Note*: Tukey multiple comparisons test (*p* value).

* Indicates *p* < 0.05.

### Mechanical strain modulates myokine secretion by muscle constructs

**Fig. 4:**
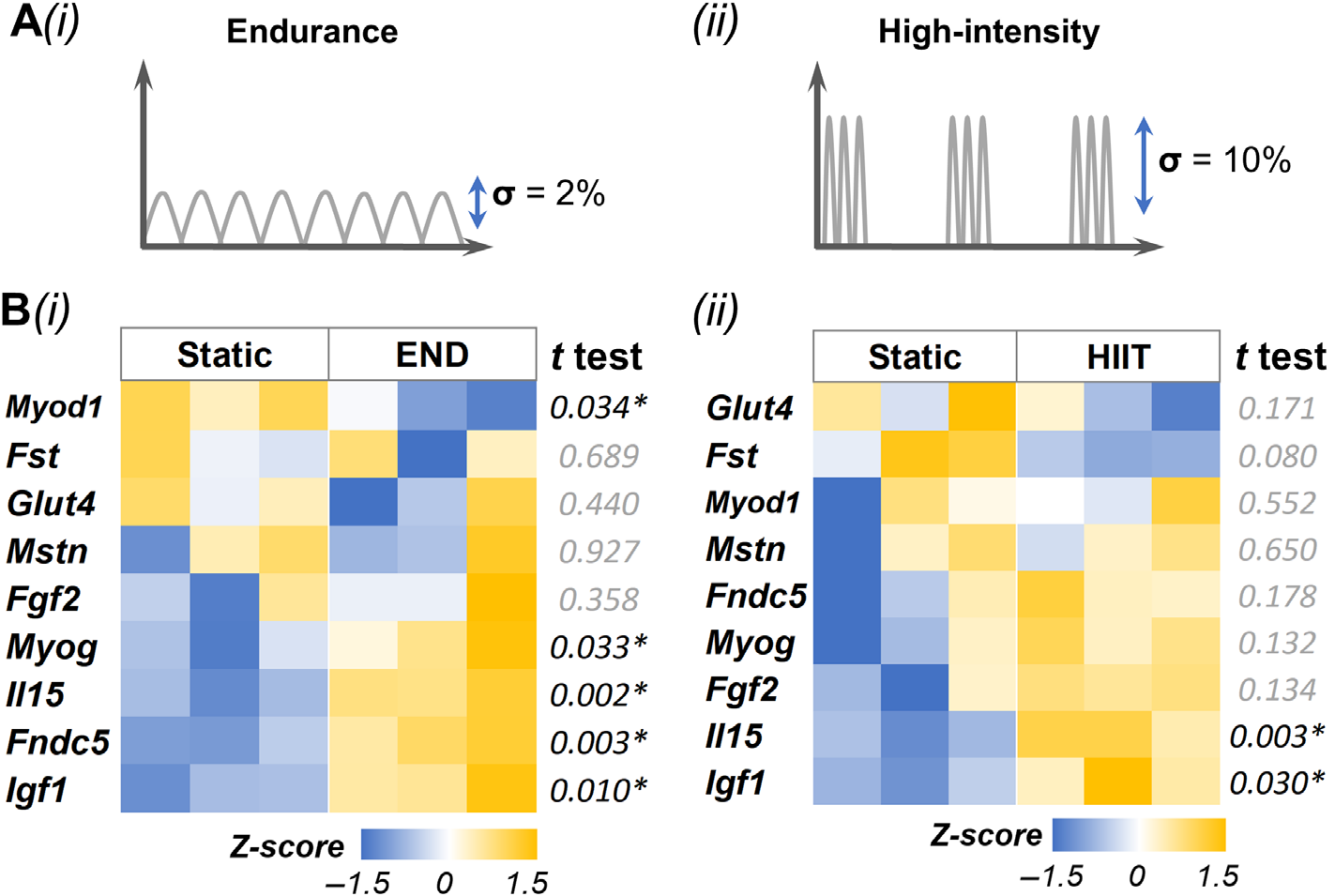
Mechanical strain modulates muscle phenotype and myokine expression. (*A*) Schematic of exercise regimen (i) endurance (END) and (ii) high‐intensity interval training (HIIT). (*B*) Gene expression results of (i) END and (ii) HIIT. All gene expressions were normalized to static control and housekeeping gene Ppia expression. *n* = 3 for each group.

We created muscle constructs in 1.2 × 1.2 cm PDMS wells and subjected them to mechanical stimulation using END and HIIT exercise regimens for 6 hours, and their phenotypes were compared with static controls (Fig. 4*A*). A qPCR analysis (Fig. 4*B*) showed that *Igf1* and *Il15* were significantly upregulated in both exercise regimens. However, in END conditions, we also noticed a significant upregulation of *Myog* and downregulation of *Myod1*, indicating differentiation of myoblasts to myotubes. In addition, the osteoinductive myokine irisin (*Fndc5*) was also upregulated in END. There was no significant difference in the expression levels of *Fst*, *Mstn*, and *Fgf2* under both exercise regimens.

**Fig. 5:**
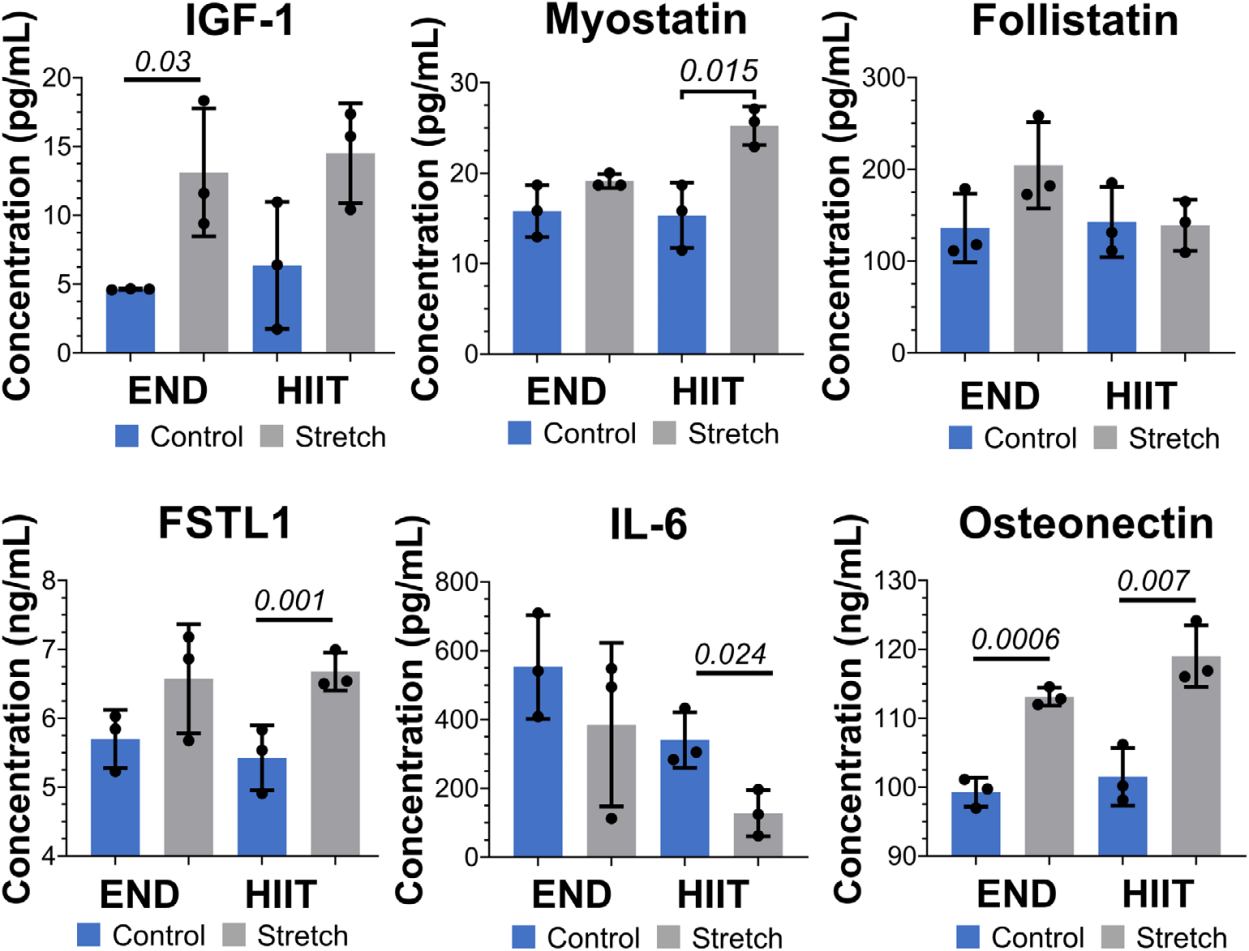
Mechanical strain modulates myokine secretion. The plots show the secretion of various myokines, insulin‐like growth factor (IGF‐1), myostatin (MSTN), follistatin (FST), follistatin‐like 1 protein (FSTL1), interleukin 6 (IL‐6), and osteonectin (SPARC) in conditioned media of muscle constructs subjected to endurance (END) or high‐intensity interval (HIIT) mechanical stimulation (stretch) compared with their static control. *n* = 3 for each group.

The concentrations of secreted myokines by muscle constructs subjected to END or HIIT stimulation were then analyzed using ELISA or multiplex kits and compared to corresponding static controls (Fig. 5). Compared with the static group, the constructs subjected to END showed significant upregulation of IGF‐1 and osteonectin (SPARC). Likewise, the constructs subjected to HIIT showed significant upregulation of myostatin, follistatin‐like 1 (FSTL‐1), and osteonectin. However, no significant change in follistatin secretion was noticed in either condition. Further, no detectable amounts of the secreted irisin (FNDC5), oncostatin M (OSM), and interleukin 15 (IL‐15) were found in the media.

### Mechanical strain modulates myokine secretion and influences bone phenotype

**Fig. 6:**
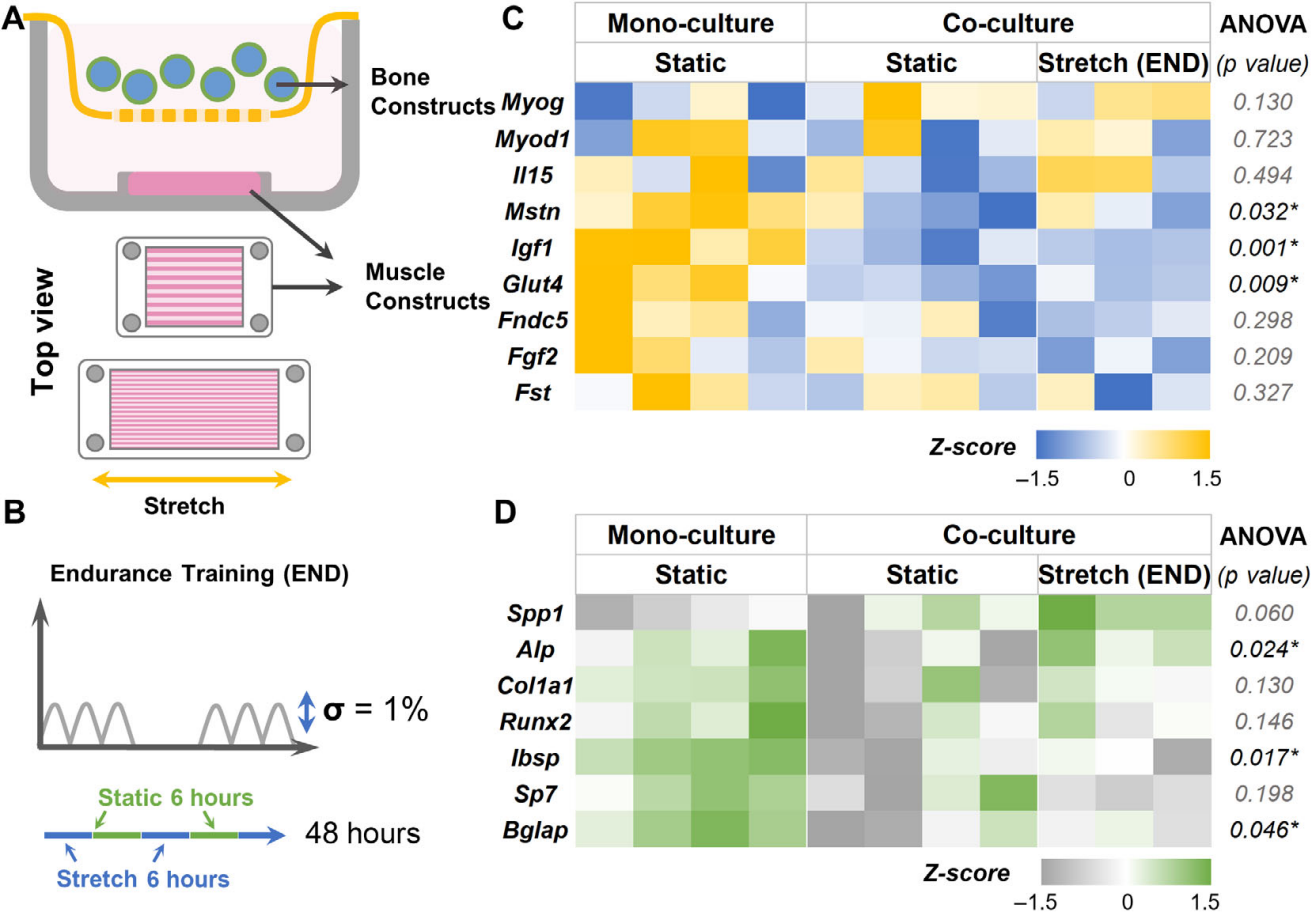
Effects of endurance training on myokine and bone phenotype. (*A*) Schematic showing the setup of the muscle‐bone construct co‐cultures. (*B*) The 48‐hour endurance (END) exercise regimen consists of alternating 6‐hour stretch (1% strain) at 1/30 Hz and 6‐hour rest. (*C*) Heat maps showing the gene expression of muscle constructs subjected to END regimen when co‐cultured with bone constructs. (*D*) Gene expression heat maps of the corresponding bone constructs in the co‐cultures. All gene expressions were normalized to monoculture controls and housekeeping genes Gapdh (bone) and Ppia (muscle). *n* = 3 for stretch groups and *n* = 4 for static groups.

We studied the effect of mechanical strain on muscle constructs when they are co‐cultured with bone constructs. We used a transwell fitted with a 100‐μm nylon mesh to separate the bone constructs from the muscle constructs while allowing only soluble factors to diffuse (Fig. 6*A*). Then, we independently subjected the muscle constructs to a linear strain. To mimic endurance training (END), the constructs were subjected to 1% strain at 1/30 Hz in bouts of 6 hours for 24 hours with 6‐hour intervals (Fig. 6*B*). Gene expression analyses showed that myostatin (*Mstn*) was significantly downregulated in co‐cultured muscle constructs along with *Igf1* and *Glut4* compared with monocultures in static conditions (Fig. 6*C*). Likewise, *Alp*, a prominent osteogenic marker, was significantly upregulated in the bone constructs co‐cultured with muscle constructs subjected to END regimen (Fig. 6*D*). But when bone constructs were co‐cultured with muscle constructs that were not stretched, we noticed significant downregulation of several osteogenic genes (see Tables 3 and 4 for muscle and bone groupwise comparison, respectively).

**Table 3:** Change in Gene Expression of Muscle Constructs Subjected to END

Genes	ANOVA	Mono vs. co‐static	Mono vs. stretch (END)	Co‐static vs. stretch (END)
*Igf1*	0.001*	0.001*	0.002*	0.965
*Mstn*	0.032*	0.028*	0.161	0.627
*Glut4*	0.009*	0.010*	0.033*	0.851
*MyoD*	0.723	0.711	0.863	0.973
*Fndc5*	0.298	0.404	0.338	0.967
*Fst*	0.327	0.664	0.301	0.726
*Fgf2*	0.209	0.523	0.189	0.659
*IL‐15*	0.494	0.694	0.905	0.485
*Myog*	0.130	0.137	0.265	0.947

*Note*: Tukey multiple comparisons test (*p* value).

Abbreviation: END = endurance training.

* Indicates *p* < 0.05.

**Table 4:** Change in Gene Expression of Bone Constructs Co‐cultured With Muscle Construct (END)

Genes	ANOVA (*p* value)	Mono vs. co‐static	Mono vs. stretch (END)	Co‐static vs. stretch (END)
*Alp*	0.024*	0.042*	0.967	0.040*
*Bglap*	0.046*	0.041*	0.231	0.610
*Ibsp*	0.017*	0.019*	0.059	0.860
*Col1a1*	0.131	0.118	0.755	0.395
*Runx2*	0.146	0.131	0.757	0.429
*Sp7*	0.199	0.376	0.200	0.831
*Spp1*	0.061	0.675	0.054	0.173

*Note*: Tukey multiple comparisons test (*p* value).

Abbreviation: END = endurance training.

* Indicates *p* < 0.05.

**Fig. 7:**
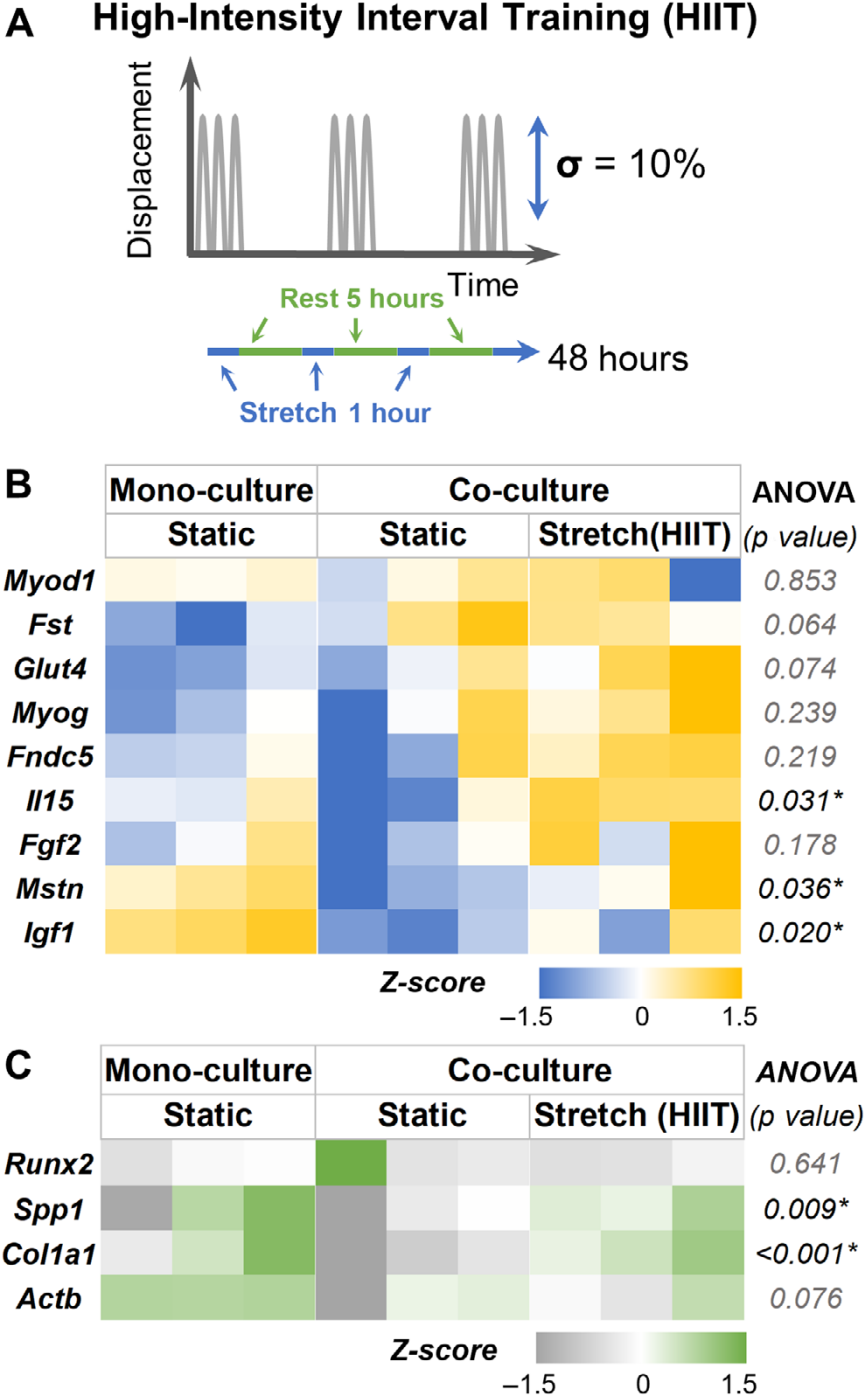
Effects of high‐intensity interval (HIIT) training on myokine and bone phenotype. (*A*) The HIIT exercise regimen consists of 10% strain at 1/30 Hz in bouts of 1 for 8 hours with 5‐hour intervals. (*B*) Heat maps showing the gene expression of muscle constructs subjected to HIIT training when co‐cultured with bone constructs. (*C*) Gene expression heat maps of the corresponding bone constructs in the co‐cultures. All gene expressions were normalized to monoculture controls and housekeeping genes Gapdh (bone) and Ppia (muscle). *n* = 3 for all groups.

We then investigated the effect of the HIIT exercise regimen (Fig. 7*A*) and noticed significant downregulation in *Mstn* in co‐cultured muscle constructs similar to END regimen. To mimic HIIT, the constructs were subjected to 10% strain at 1/30 Hz in bouts of 1 hour for 8 hours with 6‐hour intervals (Fig. 7*B*). Interestingly, expression of *Il15* was significantly increased (*p* = 0.026) in muscle constructs subjected to HIIT exercise regimen compared with static condition. Further, *Glut4* expression did not change significantly (*p* = 0.06) in the co‐cultured muscle constructs compared with static monocultures (Fig. 7*C*; see Table 5 for groupwise comparison). The altered myokine expression also altered the expression of osteogenic genes in co‐cultured bone constructs (Fig. 7*D*). There was a significant upregulation of genes, including *Spp1* and *Col1a1*, in the co‐cultured bone constructs compared with static controls (see Table 6 for groupwise comparison).

**Table 5:** Change in Gene Expression of Muscle Constructs Subjected to HIIT

Genes	ANOVA (*p* value)	Mono vs. co‐static	Mono vs. stretch (HIIT)	Co‐static vs. stretch (HIIT)
*Igf1*	0.020*	0.017*	0.188	0.203
*Mstn*	0.036*	0.046*	0.966	0.063
*Il15*	0.031*	0.237	0.256	0.026*
*Fst*	0.064	0.079	0.103	0.976
*Glut4*	0.074	0.545	0.065	0.263
*Myog*	0.239	0.850	0.229	0.439
*Myod1*	0.853	0.999	0.866	0.889
*Fndc5*	0.219	0.940	0.343	0.228
*Fgf2*	0.178	0.562	0.555	0.157

*Note*: Tukey multiple comparisons test (*p* value).

Abbreviation: HIIT = high‐intensity interval training.

* Indicates *p* < 0.05.

**Table 6:** Change in Gene Expression of Bone Constructs Co‐cultured With Muscle Construct (HIIT)

Genes	ANOVA (*p* value)	Mono vs. co‐static	Mono vs. stretch (HIIT)	Co‐static vs. stretch (HIIT)
*Col1a1*	<0.001*	0.009*	0.013*	<0.001*
*Spp1*	0.01*	0.355	0.049*	0.008*
*Actb*	0.643	0.647	0.980	0.753
*Runx2*	0.076	0.405	0.469	0.066

*Note*: Tukey multiple comparisons test (*p* value).

Abbreviation: HIIT = high‐intensity interval training.

* Indicates *p* < 0.05.

### Myostatin and follistatin influence osteogenesis of progenitor cells

**Fig. 8:**
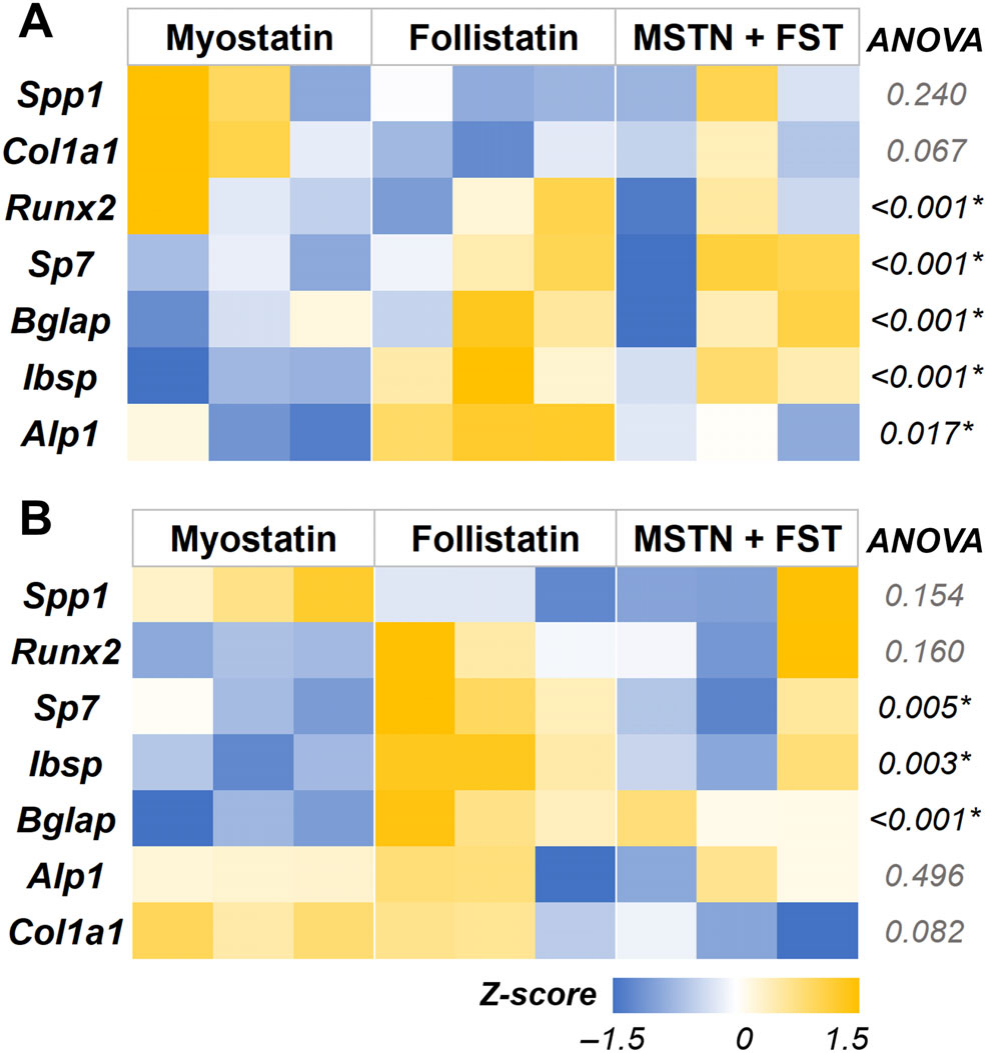
Myostatin treatment negatively regulated prominent osteogenic gene expression. Heat maps showing the osteogenic gene expression of preosteoblast cells cultured in growth media supplemented with myostatin, follistatin, or a combination of both for (*A*) 48 hours and (*B*) 7 days. MSTN = myostatin; FST = follistatin. All gene expressions were normalized to no‐treatment control and housekeeping gene Gapdh expression. *n* = 3 for all groups.

From the above studies, the myokines expressed under the HIIT regimen seem to promote a healthier bone phenotype than END training in hyperglycemia. But it is also possible that the degree to which *Mstn* is expressed or suppressed in muscle constructs primarily determines the beneficial effect on bone phenotype. Since measuring the secreted protein concentrations was challenging in co‐culture conditions, we used recombinant myostatin and follistatin to validate their role in the osteogenic differentiation of preosteoblasts. The preosteoblasts were cultured in both growth and osteogenic conditions. The media was supplemented with myostatin (100 ng/mL), follistatin (100 ng/mL), or a combination of both (100 ng/mL each). In growth conditions, the myostatin treatment significantly downregulated the expression of osteogenic genes *Ibsp*, *Bglap*, *Sp7*, *Alp1*, and *Runx2* within 48 hours of treatment (Fig. 8*A*; see Table 7 for groupwise comparison). Likewise, when follistatin was supplemented in the growth media, there was a significant downregulation of osteogenic genes *Bglap*, *Ibsp*, and *Runx2* within 48 hours of treatment. But when compared with the myostatin treatment group, the follistatin treatment significantly upregulated *Ibsp* and *Alp1* expression. When both myostatin and follistatin were supplemented together in the media, there was a significant downregulation of *Bglap*, *Ibsp*, *Alp1*, and *Runx2*. In long‐term cultures (7 days; Fig. 8*B*; see Table 8 for groupwise comparison), the myostatin treatment significantly downregulated the expression of *Bglap*, *Ibsp*, and *Sp7*, similar to the short‐term effects. However, follistatin treatment significantly upregulated the osteogenic genes *Bglap*, *Ibsp*, and *Sp7* in long‐term cultures. When both myostatin and follistatin were added, there was a significant downregulation of *Bglap, Ibsp*, and *Sp7* compared with the control group. Nevertheless, when compared with the myostatin treatment group, the combinatorial treatment significantly upregulated *Bglap*, and there was no change in the expression levels of other osteogenic genes. No significant changes were found between treatment groups when the constructs were cultured in osteogenic media (Supplemental Fig. S2).

**Table 7:** Gene Expression of Osteoblasts Treated With Myostatin and Follistatin for 48 Hours

Genes	ANOVA (*p* value)	Control vs. MSTN	Control vs. FST	Control vs. MSTN + FST	MSTN vs. FST	MSTN vs. MSTN + FST	FST vs. MSTN + FST
*Bglap*	0.010*	0.012*	0.045*	0.022*	0.771	0.971	0.948
*Ibsp*	<0.001*	<0.001*	0.015*	0.005*	0.022*	0.078	0.802
*Alp1*	<0.001*	0.001*	0.101	0.001*	0.012*	0.805	0.042*
*Sp7*	0.017*	0.015*	0.100	0.056	0.540	0.768	0.975
*Runx2*	<0.001*	0.002*	0.001*	0.001*	0.988	0.778	0.918
*Spp1*	0.240	0.787	0.214	0.438	0.630	0.914	0.936
*Col1a1*	0.066	0.997	0.125	0.374	0.095	0.295	0.838
*Act1*	0.821	0.851	0.999	0.918	0.900	0.998	0.952

*Note*: Tukey multiple comparisons test (*p* value).

Abbreviation: FST = follistatin; MSTN = myostatin.

* Indicates *p* < 0.05.

**Table 8:** Gene Expression of Osteoblasts Treated With Myostatin and Follistatin for 7 Days

Genes	ANOVA (*p* value)	Control vs. MSTN	Control vs. FST	Control vs. MSTN + FST	MSTN vs. FST	MSTN vs. MSTN + FST	FST vs. MSTN + FST
*Alp1*	0.496	0.816	0.479	0.609	0.922	0.979	0.995
*Col1a1*	0.082	0.888	0.999	0.193	0.827	0.071	0.233
*Runx2*	0.160	0.155	0.983	0.699	0.251	0.591	0.876
*Bglap*	<0.001*	<0.001*	0.119	0.017*	0.002*	0.010*	0.553
*Spp1*	0.154	0.132	0.922	0.631	0.305	0.594	0.930
*Ibsp*	0.003*	0.005*	0.914	0.038*	0.012*	0.487	0.097
*Sp7*	0.005*	0.009*	0.529	0.013*	0.067	0.995	0.094

*Note*: Tukey multiple comparisons test (*p* value).

Abbreviation: FST = follistatin; MSTN = myostatin.

* Indicates *p* < 0.05.

## Discussion

Uncoupling the effects of biophysical and biochemical stimuli on the adaptive response of bone during exercise training is challenging. We addressed this limitation by designing an in vitro co‐culture system that allows independent mechanical manipulation of bone and muscle constructs. The muscle constructs were created in a stretchable PDMS well by seeding murine myoblasts in fibrin hydrogels that formed multinucleated myotubes upon serum starvation. The increase in *Myog* and *Myod* expression levels are indicators of myoblast differentiation into myotube.^(^ ^40^ ^)^ Early *Myod* expression is essential for myoblast proliferation and induction of differentiation, whereas the late expression of *Myog* is vital for the terminal differentiation and fusion of myoblast into mature muscle fibers.^(^ ^41^ ^)^ Fibrin hydrogels allowed modulating mechanical properties, including stiffness and pore size, and favored myogenesis by supporting myoblast proliferation and myotube formation.^(^ ^42^ ^)^ On the other hand, the cross‐linked gelatin microgels supported the osteogenic differentiation of progenitor cells. The physical properties, including the curvature, stiffness, and surface characteristics of the microgels, contributed significantly to its osteoconductive property.^(^ ^34^, ^43^, ^44^ ^)^ Using these constructs, we evaluated the effects of mechanical strain on myokine secretion under hyperglycemic conditions and its impact on bone metabolism decoupled from physical stimuli.

Exercise training has been shown to have protective effects on muscle and bone biology. Exercise training can activate resident myogenic stem cells, also known as satellite cells, to produce hepatocyte growth factor (HGF) that can promote myogenesis, muscle repair, and regeneration.^(^ ^45^ ^)^ In addition, exercise training influences the cellular metabolism of muscles, including glucose uptake and phosphorylation of anabolic targets.^(^ ^46^ ^)^ This is particularly relevant for studying how skeletal muscle responds to these exercise‐induced physiologic changes in a diabetic environment. Our studies show that in hyperglycemic conditions, both HIIT and END exercise regimens increase the expression levels of *Igf1* and *Il15*. But a corresponding increase in secreted IGF‐1 protein was found only in END media, and IL15 was not detected in either condition. Likewise, a significant increase in the secretion of osteonectin (SPARC), a protein that can promote mineralization of the bone constructs, was found in both conditions.^(^ ^47^ ^)^ In addition, HIIT can also increase myostatin (MSTN) secretion, which could be detrimental to the osteogenic phenotype. On the other hand, END strain regimen seems to contribute significantly to the differentiation of myoblasts, as evident from the upregulation of *Myog* and downregulation of *Myod1*. The osteoinductive myokine irisin (*Fndc5*) was also significantly upregulated in END, indicating that END regimen may be more suitable for increasing bone quality and density^(^ ^48^ ^)^ while contributing to muscle hypertrophy.^(^ ^48^, ^49^ ^)^

Interestingly, when muscle constructs are co‐cultured with bone constructs, the effect of the exercise regimens changed markedly. The modular nature of the constructs enabled co‐culturing the muscle constructs with bone constructs in a biomechanically decoupled fashion. Under static conditions, there was an upregulation of *Myog* and *Fst* in muscle constructs and *Spp1* (osteopontin) in bone constructs. Osteopontin is a matricellular protein that promotes proliferation and calcification in osteoblasts and mediates bone metabolism.^(^ ^50^ ^)^ Further, *Mstn* expression was significantly downregulated in muscle constructs co‐cultured with bone constructs compared with monocultures. Myostatin can bind to activin receptor IIB (ActRIIB) in osteogenic cells and negatively regulate osteoblast and osteoclast gene expression.^(^ ^51^ ^)^ The role of myostatin in skeletal muscle is redundant with another protein of the TGFβ family, activin A. But inhibiting myostatin activity in muscles improves muscle health and promotes muscle hypertrophy.^(^ ^52^ ^)^ Follistatin, an endogenous inhibitor of myostatin, is also shown to promote muscle growth.^(^ ^32^ ^)^ Hence, in a hyperglycemic environment and in the absence of biomechanical stimulation, muscle‐bone biochemical interaction results in alterations of gene transcripts of important myokines, such as myostatin and follistatin, that facilitate muscle‐bone communication.

When muscle constructs were independently subjected to mechanical stimulation in co‐cultures, we saw muscle and bone phenotype changes depending on the exercise regimen. Notably, *Igf1* was significantly downregulated in co‐cultured muscle constructs subjected to END but not in HIIT. Consequently, the insulin/IGF1‐regulated glucose transporter, *Glut4*, was significantly downregulated in co‐cultured muscle construct under END regimen but upregulated in the HIIT. Studies show that even in non‐diabetic conditions, endurance training can reduce muscle glucose uptake due to reduced *GLUT4* translocation,^(^ ^53^, ^54^ ^)^ whereas acute bouts of moderate‐high‐intensity exercise can increase their translocation in an insulin‐independent manner.^(^ ^55^ ^)^ Our studies also showed a similar exercise‐dependent *GLUT4* translocation even under hyperglycemic conditions. The co‐cultured bone constructs also showed variable phenotype changes when the muscle constructs underwent mechanical strain. An early osteogenic marker, *Alp*, was significantly upregulated in END regimen, while intermediate and late‐stage osteogenic markers, *Col1a1* and *Spp1*, respectively, were significantly upregulated in the HIIT regimen. Hence, the myokines secreted under the HIIT regimen compared with END training in hyperglycemia appear to have differing effects on bone cell homeostasis. It is possible that the degree to which *Mstn* is expressed or suppressed in muscle constructs primarily determines the beneficial effect on bone phenotype. Studies show that decoy receptors of myostatin enhance the osteogenesis of progenitors^(^ ^56^ ^)^ and consequently increase bone mass and density.^(^ ^57^ ^)^ With these studies in mind, and our findings that myostatin mRNA was significantly downregulated in our co‐culture conditions, we analyzed the role of myostatin and follistatin on the phenotype of the bone constructs isolated from other myokines in hyperglycemia. Our in vitro studies confirmed the adverse effects of myostatin on bone constructs by significantly reducing osteogenic gene expressions of the osteoprogenitor cells. When we treated the bone constructs with myostatin and follistatin, we noticed that myostatin's inhibitory effects on osteogenic differentiation significantly ameliorated. More mechanistic studies using selective inhibitors of osteokines and myokines could further clarify their specific roles in physiology and pathologies, which is a limitation of our study. Further, we did not incorporate osteocytes and osteoclasts in our bone constructs, which may be a less precise representation of the in vivo bone environment. Furthermore, exploring the application of various types of matrices, such as cross‐linked collagen, to mimic the bone and muscle phenotype would be beneficial and will be the primary focus of our future work.

Together, our in vitro co‐culture system was able to elucidate the effects of different strain regimens on myokine secretion and its impact on bone metabolism in hyperglycemia, mimicking a diabetic environment. END strain regimen seems to contribute more significantly to the differentiation of myoblasts than HIIT. But when co‐cultured with bone constructs, the myokine expression in the HIIT regimen is more likely to enhance muscle phenotype. Likewise, the myokines secreted under the HIIT regimen promote a healthier bone phenotype than END training. Together, our in vitro studies suggest that under hyperglycemia, the type of exercise influences muscle phenotype, and the secreted biochemical signals from muscle and bone constructs can influence each other, even in the absence of biomechanical stimuli.

Overall, our co‐culture system allowed orthogonal manipulation of mechanical strain on muscle constructs while facilitating biochemical cross‐talk between bone and muscle constructs. In addition, our system is innovative, as it provides an individualized microenvironment and allows decoupled biomechanical manipulation, which is unachievable using traditional models. In the long term, these in vitro systems can help identify molecular targets and develop engineered therapies for diabetic bone disease.

## Author Contributions

**Harshini Suresh Kumar:** Data curation; formal analysis; investigation; writing – review and editing. **Edwina N. Barnett:** Data curation; writing – review and editing. **John L. Fowlkes:** Supervision; writing – review and editing. **Evangelia Kalaitzoglou:** Conceptualization; formal analysis; funding acquisition; investigation; methodology; project administration; resources; validation; writing – review and editing. **Ramkumar T. Annamalai:** Conceptualization; data curation; formal analysis; funding acquisition; investigation; methodology; project administration; resources; software; supervision; validation; visualization; writing – original draft; writing – review and editing.

## Disclosures

The authors declare no conflicts of interest.

### Peer Review

The peer review history for this article is available at https://www.webofscience.com/api/gateway/wos/peer‐review/10.1002/jbm4.10804.

## Supporting information


**Figure S1.** Heatmaps depicting the osteogenic gene expression of cells seeded on cross‐linked gelatin microgels and cultured in growth media. *n* = 3 for all groups.
**Figure S2.** Heatmaps depicting the osteogenic gene expression of cells cultured in osteogenic media supplemented with myostatin, follistatin, or a combination of both. *n* = 3 for all groups.

## Data Availability

All data that support the findings of this study are presented in the main article or in the supplemental section.
