# Supplementary material for: Biomechanical Stimulation of Muscle Constructs Influences Phenotype of Bone Constructs by Modulating Myokine Secretion
Source: JBMR Plus. 2023 Aug 15;7(11):e10804. doi: 10.1002/jbm4.10804 (PMC10652181; doi:10.1002/jbm4.10804)
Supplement: Supplementary file 1 — Figure S1. Heatmaps depicting the osteogenic gene expression of cells seeded on cross‐linked gelatin microgels and cultured in growth media. n = 3 for all groups. Figure S2. Heatmaps depicting the osteogenic gene expression of cells cultured in osteogenic media supplemented with myostatin, follistatin, or a combination of both. n = 3 for all groups. [file JBM4-7-e10804-s001.docx]

# Supplementary information


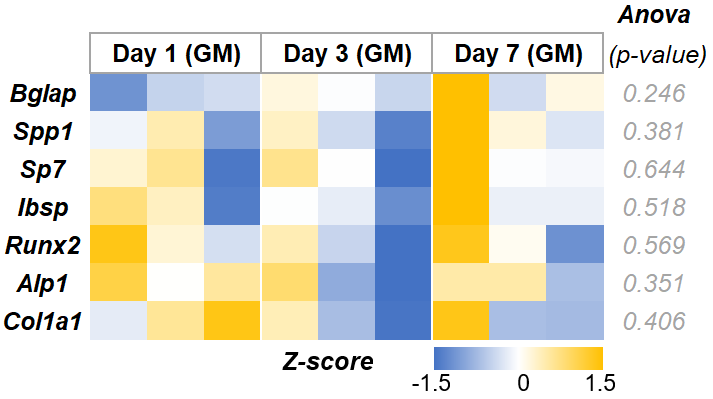


***Supplementary Figure 1****: Heatmaps depicting the osteogenic gene expression of cells seeded on cross-linked gelatin microgels and cultured in growth media. n=3 for all groups.*

*
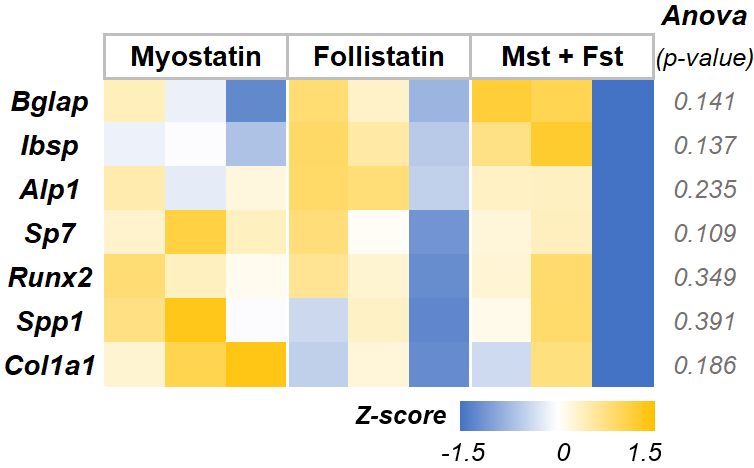
*

***Supplementary Figure 2****: Heatmaps depicting the osteogenic gene expression of cells cultured in osteogenic media supplemented with myostatin, follistatin, or a combination of both. n=3 for all groups.*
